# Five-Year Outcomes After Endovascular Treatment for Large Vessel Occlusion Stroke

**DOI:** 10.3389/fnins.2022.920731

**Published:** 2022-07-13

**Authors:** Changxiong Gong, Jiacheng Huang, Weilin Kong, Fengli Li, Chang Liu, Jie Yang, Shuai Liu, Zhongming Qiu, Min Lin, Zhangbao Guo, Zhizhong Yan, Xianjun Huang, Shuai Zhang, Wentong Ling, Peiyang Zhou, Zhen Wang, Yong Liu, Dongzhang Xue, Yaoyi Zhong, Shu Yang, Yue Wan, Jiayang Fang, Wenguo Huang, Huihui Liu, Jun Luo, Rongzhong Li, Changming Wen, Xinmin Fu, Mingyi Tu, Li Wang, Xiguang Tian, Huiyuan Peng, Zhilin Wu, Guoyong Zeng, Wenjie Zi, Qingwu Yang

**Affiliations:** ^1^Department of Neurology, Xinqiao Hospital and The Second Affiliated Hospital, Army Medical University (Third Military Medical University), Chongqing, China; ^2^Department of Neurology, The Second Affiliated Hospital and Fujian University of Traditional Chinese Medicine, Fuzhou, China; ^3^Department of Neurology, The 900th Hospital of The People’s Liberation Army, Fuzhou, China; ^4^Department of Neurology, Wuhan No. 1 Hospital, Wuhan, China; ^5^Department of Neurosurgery, The 904th Hospital of The People’s Liberation Army, Wuxi, China; ^6^Department of Neurology, Yijishan Hospital of Wannan Medical College, Wuhu, China; ^7^Department of Neurology, The Affiliated Hospital of Yangzhou University, Yangzhou, China; ^8^Department of Neurology, Zhongshan People’s Hospital, Zhongshan, China; ^9^Department of Neurology, The First People’s Hospital of Xiangyang and Hubei Medical University, Xiangyang, China; ^10^Department of Neurology, Changsha Central Hospital, Changsha, China; ^11^Department of Neurology, Lu’an Affiliated Hospital of Anhui Medical University, Lu’an, China; ^12^Department of Neurology, The 902th Hospital of The People’s Liberation Army, Bengbu, China; ^13^Department of Neurology, The 175th Hospital of The People’s Liberation Army, Zhangzhou, China; ^14^Department of Neurology, Sichuan Provincial People’s Hospital, Chengdu, China; ^15^Department of Neurology, Hubei Zhongshan Hospital, Wuhan, China; ^16^Department of Neurology, The 476th Hospital of The People’s Liberation Army, Fuzhou, China; ^17^Department of Neurology, Chinese Medical Hospital of Maoming, Maoming, China; ^18^Department of Neurology and Suzhou Clinical Research Center of Neurological Disease, Second Affiliated Hospital of Soochow, Suzhou, China; ^19^Department of Neurology, Sichuan Mianyang 404 Hospital, Mianyang, China; ^20^Department of Neurology, The 924th Hospital of The People’s Liberation Army, Guilin, China; ^21^Department of Neurology, Nanyang Central Hospital, Nanyang, China; ^22^Department of Neurology, Xuzhou Central Hospital, Xuzhou, China; ^23^Department of Neurology, Hubei Wuchang Hospital, Wuhan, China; ^24^Department of Neurology, The Third People’s Hospital of Zigong, Zigong, China; ^25^Department of Neurology, The Chinese Armed Police Force Guangdong Armed Police Corps Hospital, Guangzhou, China; ^26^Department of Neurology, Chinese Medical Hospital of Zhongshan, Zhongshan, China; ^27^Department of Neurology, Yunfu People’s Hospital, Yunfu, China; ^28^Department of Neurology, Ganzhou People’s Hospital, Ganzhou, China

**Keywords:** endovascular treatment, ischemic stroke, large vessel occlusion, long-term patient outcome, stroke recurrence

## Abstract

**Background:**

The long-term outcomes of acute large vessel occlusion (LVO) in anterior circulation treated by endovascular treatment (EVT) remains to be determined. The aim of this study was to assess the 5-year outcomes of patients with LVO who underwent EVT.

**Methods:**

This study was an observational, nationwide registry of consecutive patients with acute LVO who received EVT in 28 comprehensive stroke centers in China. The primary outcome was the proportion of favorable outcome [modified Rankin Scale score (mRS) 0–2] at 5 years. Secondary outcomes included proportions of patients with excellent outcome (mRS 0–1), all-cause mortality and risk of stroke recurrence at 5 years.

**Results:**

A total of 807 patients were included into the study and had 90-day follow-up data, 657 patients had 5-year follow-up data. At 90 days, 218 patients (27.0%) had an excellent outcome, 349 patients (43.2%) had a favorable functional outcome. 199 patients (24.7%) died. At 5 years, 190 patients (28.9%) had an excellent outcome, 261 patients (39.7%) had a favorable functional outcome, 317 patients (48.2%) died and 129 (28.2%) had stroke recurrence. Because of missing 5-year follow-up data, among available 269 patients who achieved functional independence at 90 days, 208 (77.3%) maintained favorable outcome, 19 (7.1%) had disability (mRS 3–5) and 42 (15.6%) died at 5 years. Furthermore, among available 189 patients with mRS 3–5 at 90 days, 53 (28.0%) patients achieved favorable functional outcome, 60 (31.7%) patients maintained unfavorable functional outcome and 76 (40.2%) patients died within 5 years. Multivariate analyses identified that younger age [odds ratio (OR): 0.96; 95% CI, 0.93–0.99; *P* = 0.009], lower mRS at 90 days (OR: 0.15; 95% CI, 0.10–0.23; *P* < 0.001) and absence of stroke recurrence (OR: 0.001; 95% CI, 0.000–0.006; *P* < 0.001) were significantly associated with favorable outcome at 5 years. Advanced age (OR: 1.06, 95% CI, 1.04–1.08; *P* < 0.001), higher mRS at 90 days (OR: 0.84; 95% CI, 0.73–0.98; *P* = 0.021) and atrial fibrillation (OR: 1.63; 95% CI, 1.02–2.60; *P* = 0.04) were independent factors for stroke recurrence.

**Conclusion:**

Our results indicated that the beneficial effect of EVT in patients with acute LVO can be sustained during the course of at least 5 years. Reducing the risk of stroke recurrence by anticoagulation for atrial fibrillation may be a crucial strategy to improve long-term outcome.

## Introduction

Stroke is the primary leading cause of death in China. Randomized studies have established endovascular treatment (EVT) as a standard of care for acute ischemic stroke (AIS) due to large vessel occlusion (LVO) in the anterior circulation (Berkhemer et al., 2015; Campbell et al., 2015; Goyal et al., 2015; Jovin et al., 2015; Saver et al., 2015; Bracard et al., 2016; Martins et al., 2020). Limitations of these studies and previous trials of thrombectomy with large vessel occlusion for acute ischemic stroke included the lack of long-term follow-up (McCarthy et al., 2017). Recently, follow-up outcomes from the 2-year MR CLEAN and 12-month REVASCAT trial were reported (Dávalos et al., 2017; van den Berg et al., 2017). In MR CLEAN, only 391 patients obtained extended follow-up outcomes in the initially enrolled 500 patients. After 2 years, mRS scores in the endovascular group continued to maintain significantly better than those of standard medical therapy. The researchers also performed a subgroup analysis, defining patients as those with an excellent outcome, and favorable outcome. The endovascular group was more likely to have a favorable outcome, but no difference was noted in excellent outcomes (van den Berg et al., 2017). The REVASCAT outcomes in 12-month included 205 of the initial 206 patients (99%). At 12-months, mRS scores continued to favor endovascular treatment. Initial 90-day functional independence benefit seen with endovascular therapy was maintained at 12-months (Dávalos et al., 2017). Both studies showed a statistically significant clinical benefit for mechanical thrombectomy with regards to quality of life, measured with EuroQol- 5 Dimension (EQ-5D), at the extended follow-up. A large number of patients with mechanical thrombectomy have a favorable long-term outcome. Outcomes remained stable between short- and long-term follow-up, but some individuals may still show improvement beyond short-term rehabilitation (Fuhrer et al., 2020). A meta-analysis concluded that endovascular therapy results in favorable outcomes at long-term follow-up for patients with acute ischemic stroke compared to standard medical treatment alone and that the 90-day timepoint offers a fair representation of the long-term outcomes (McCarthy et al., 2019). Recently, the data from real-world studies with large sample have shown that patients treated with endovascular therapy had longer survival times, attributable a lower risk of death within 0–90 days and extended follow-up to 2 years (Beyeler et al., 2022). Despite these promising finding, much of our understanding about stroke outcomes comes from studies with relatively short follow-up time and the long-term prognosis after a stroke are sparse (Rutten-Jacobs et al., 2013; Ekker et al., 2019; Peng et al., 2022). Acute stroke trials have largely assessed short-term (90 days) primary outcomes following treatment (Yarbrough et al., 2015; Campbell et al., 2016; Goyal et al., 2016) and information on long-term outcomes may be useful for clinical practice and health care policy decision-making, particularly in developing countries which have a higher stroke burden, but more limited health care resources. The long-term (5 or more years) outcomes after stroke have received less attention. To our knowledge, only a few studies reported secondary outcomes up to or longer than 2 years (Palesch et al., 2015; Dávalos et al., 2017; van den Berg et al., 2017). Hence, long-term functional outcomes, stroke recurrence and mortality of patients with acute LVO in anterior circulation are largely uncertain.

The endovascular treatment for acute ischemic stroke due to large vessel occlusion in the anterior circulation registry (SUSTAIN) aims to estimate the effect of EVT on functional outcome at 5 years in a real-world setting.

## Materials and Methods

The data supporting the findings in this study are available from the corresponding author upon reasonable request.

### Study Design

SUSTAIN was an observational, nationwide registry of consecutive patients aged 18 years or older who presented with acute anterior circulation stroke in whom an EVT procedure was attempted for LVO in 28 comprehensive stroke centers across 12 provinces in China. The present registry was launched in 2015 aimed to determine the long-term (5-year) outcomes. Participating centers were allowed to retrospectively include patients in the registry if patients were treated within the past 3 years and if reliable clinical follow up information was available. To avoid selection bias, all participating centers were obliged to enroll all consecutive patients in the registry, including those being retrospectively enrolled in the database and those with unsuccessful treatment. All participating centers were monitored at the end of the recruitment period in order to avoid selection bias by cross-checking neurointerventional logs with the database. To be fully eligible for participation in the registry, study site selection had to meet the following minimum criteria: (1) all study centers were required to have performed at least 30 endovascular procedures annually, including at least 15 thrombectomy surgeries with the stent–retrieval devices; (2) all intervention teams were certified interventionists for intra–arterial intervention on anterior circulation large–artery occlusion.

The study protocol was approved by medical ethics committee of the Second Affiliated Hospital of the Army Medical University and all participating centers. Written informed consent was obtained from the patient or patient’s representative, as required by national and local guidelines.

### Patient Selection

In this study, the selection criteria for MT were to the discretion of the treating neuroradiologist in every center. We included data of consecutive patients with AIS if they fulfilled the following criteria: (1) an age 18 years or older; (2) diagnosed with AIS; (3) LVO in anterior circulation confirmed by computed tomographic angiography, magnetic resonance angiography, or digital subtraction angiography; (4) underwent endovascular recanalization treatment.

To keep the homogeneity of the enrolled patients, we excluded patients as the following criteria: (1) clinically significant preexisting disability with a modified Rankin Scale (mRS) score greater than 2; (2) neuroimaging evidence of cerebral hemorrhage on presentation; (3) lack of follow-up information on outcomes; (4) current pregnancy or lactation; (5) other serious, advanced, or terminal illness; (6) incomplete baseline imaging and time-metric data.

### Procedures

EVT consisted of mechanical thrombectomy, thrombo-aspiration, balloon dilation, stenting, intra-arterial thrombolysis, or various combinations of these approaches. Re-occlusion often occurred after thrombectomy in atherosclerotic disease, therefore, rescue therapy including balloon dilation, stenting, intra-arterial thrombolysis, and glycoprotein IIb/IIIa inhibitor might be utilized to retrieve recanalization. After recanalization of the target artery, most of the patients were transferred to the neuro-intensive care unit for at least 24 h with their systolic blood pressure maintained at 120–140 mmHg. Additionally, the patients who underwent extracranial or intracranial stent implantation were prescribed antithrombotic medication to prevent acute stent thrombosis. For the patients without prior intravenous alteplase, loading doses of clopidogrel (300 mg) and aspirin (300 mg) were given, or a low dose of glycoprotein IIb/IIIa inhibitor was bolus-injected intra-arterially and maintained for at least 24 h, while for those with prior intravenous alteplase, clopidogrel (75 mg) and aspirin (100 mg) were given after 24 h of alteplase administration, then all the patients were given clopidogrel (75 mg/d) and aspirin (100 mg/d) for 1–90 days (Zhuang et al., 2014).

### Data Collection

We recorded patients’ baseline characteristics, stroke risk factors, time of symptom onset, clinical presentation, stroke severity assessed by the National Institutes of Health Stroke Scale (NIHSS), pretreatment and posttreatment imaging findings, type and time of EVT, complications, presumed cause of stroke, cause of death, outcome measures, and safety measures. The presumed stroke causative mechanism was assessed based on the Trial of ORG 10,172 in Acute Stroke Treatment (TOAST) classification (Adams et al., 1993).

### Outcome Measures

The primary outcome measure was the proportion of patients with good clinical outcome at 5 years after stroke as assessed by trained local neurologists who were blinded to all the clinical data. The mRS is a 7-level scale [range, 0 (no symptoms) to 6 (death)] for the assessment of neurologic functional disability (van Swieten et al., 1988). Good clinical outcome was defined as an mRS score of 0, 1, or 2.

Secondary outcomes included the proportion of patients with excellent clinical outcome (mRS 0–1) at 5 years; death from any cause during the study period after stroke; risk of stroke recurrence that occurred within 5 years after stroke. The definition of stroke recurrence was not strictly identical in each study. In the present study, recurrent stroke was defined according to the criteria of MR CLEAN (Multicenter Randomized Clinical Trial of Endovascular Treatment for Acute Ischemic Stroke in the Netherlands) extended follow-up trial. Stroke was defined according to the World Health Organization criteria as “rapidly developing symptoms and/or signs of focal, and at times global, loss of cerebral function, with symptoms lasting more than 24 h or leading to death with no apparent cause other than that of vascular origin.” (Hatano, 1976) Although the definition of stroke recurrence was not strictly identical in each study, in practice, we used the above definition to define recurrence as stroke and met the following criteria: there is clinical evidence of the sudden onset of a new focal neurological deficit with no apparent cause other than that of vascular origin (i.e., the deficit could not be ascribed to an inter-current acute illness, epileptic seizure, or toxic effect) occurring at any time after the index stroke; or there was clinical evidence of the sudden onset of an exacerbation of a previous focal neurological deficit with no apparent cause other than that of vascular origin (Dennis et al., 1993; Burn et al., 1994; Hankey et al., 1998). Followed up was centralized and performed by trained interviewers or local neurologists and was based on a standardized interview protocol, supplemented by medical record review through hospitalization registration and video telephony or telephone interview. The outcomes of patients were investigated based on their medical records or questionnaires from patients or their relatives during the follow-up period.

### Statistical Analysis

The proportion of patients with different outcomes at 5 years was estimated after eliminating missing patients without multiple imputation. Multivariable logistic regression analysis was performed to identify factors which were associated with good outcome, mortality and stroke recurrence at 5 years in surviving patients in the periods of 90-day follow up.

Mean (standard deviation) or median [interquartile range (IQR)] was used to describe patients’ characteristics for continuous variables. Categorical variables were described as frequencies (percentages). Binary logistic regression with the enter method [ordinal variables were compared using simple (first) method] was used to identify independent predictors for poor functional outcome. Multivariate Cox regression analysis was used to determine the variables associated with higher risks of mortality and stroke recurrence at 5 years. A two-sided *P*-value of 0.05 or less was considered statistically significant. Statistical analyses were performed using SPSS version 23 (IBM Corp., Armonk, NY), R version 3.2 (R Foundation for Statistical Computing, Vienna, Austria) and STATA version 15.2 (Stata Corp LLC, TX).

## Results

### Baseline Characteristics

**FIGURE 1:**
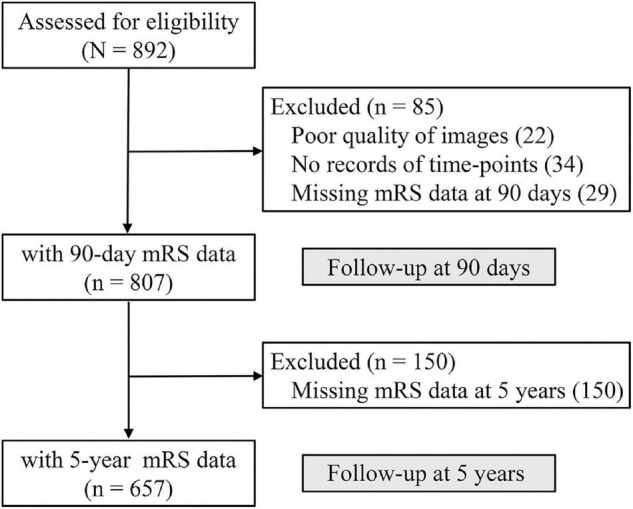
Flow diagram of the study.

The present registry opened on 1 January 2015 and is still recruiting patients. The current analysis comprises patients who entered in the database until 30 November 2016. According to the inclusion and exclusion criteria, we initially assessed 892 patients from 28 centers in China during the study period. As Figure 1 shows, 56 patients had missing critical baseline data (34 without records of time points, 22 with poor quality of images), 29 survivors without 90 days mRS, and the remaining 807 patients constituted the study population. 104 of 807 patients (12.9%) were treated before the start of the registry and were retrospectively entered in the database, and 263 (32.6%) patients received intravenous thrombolytic therapy. 657 (79.6%) had 5-year follow-up data, and 217 (33.1%) patients received intravenous thrombolytic therapy. Baseline characteristics are summarized in Table 1.

**TABLE 1:** Baseline characteristics in the study cohort.

	At 90 days (*n* = 807)	At 5 years (*n* = 657)
Age, median (IQR), y	66 (57–74)	66 (58–75)
Male, n (%)	486.807 (60.2)	387/657 (58.9)
**History, n (%)**		
Smoking	226/807 (28.0)	176/657 (26.8)
Hypertension	500/807 (62.0)	414/657 (63.0)
Hyperlipidemia	72/807 (8.9)	62/657 (9.4)
Diabetes mellitus	146/807 (18.1)	119/657 (18.1)
Atrial fibrillation	331/806 (41.1)	285/656 (43.4)
**Stroke classification, n (%)**		
Large artery atherosclerosis	359/807 (44.5)	280/657 (42.6)
Cardioembolism	389/807 (48.2)	331/657 (50.4)
Artery dissection	32/807 (4.0)	23/657 (3.5)
Other causes	27/807 (3.3)	23/657 (3.5)
**Intracranial arterial occlusion**		
Intracranial ICA	72/807 (8.9)	58/657 (8.8)
ICA T-occlusion	189/807 (23.4)	158/657 (24.0)
M1 middle cerebral artery segment	481/807 (59.6)	385/657 (58.6)
M2 middle cerebral artery segment	58/807 (7.2)	51 (7.8)
ACA	7/807 (0.9)	5/657 (0.8)
Intravenous thrombolysis	263/806 (32.6)	217/656 (33.1)
Onset to puncture time	275 (207–354)	270 (205–350)
Puncture to recanalization time	99 (75–140)	99 (75–140)
**mTICI n (%)**		
0–2a	130/803 (16.2)	107/655 (16.3)
2b–3	673/803 (83.8)	548/655 (83.7)

*IQR, interquartile range; mTICI, modified Thrombolysis in Cerebral Infarction.*

### Primary Outcome

**FIGURE 2:**
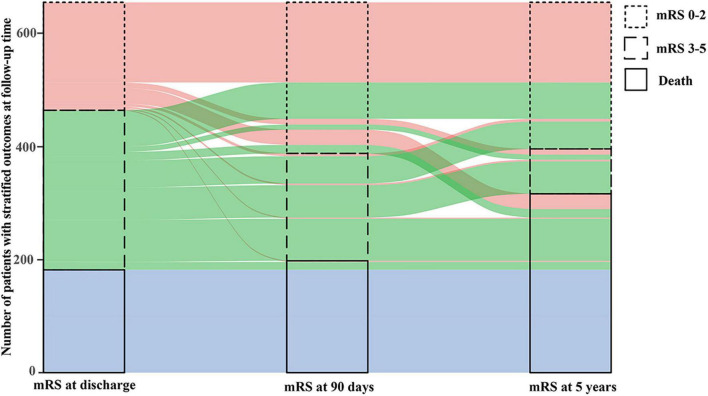
Transformation of outcomes at different follow-up time.

**FIGURE 3:**
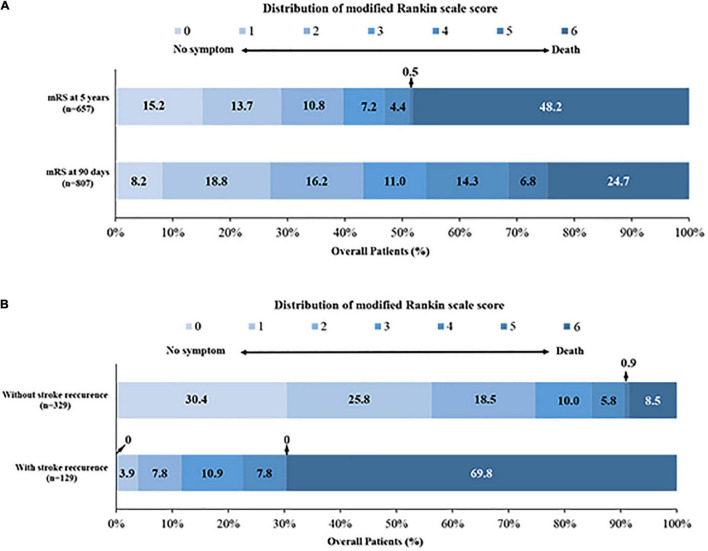
Distribution of the modified Rankin scale score. **(A)** The distribution of mRS at 90 days and 5 years; **(B)** the distribution of mRS in patients with and without stroke recurrence at 5 years.

Figure 2 shows the transformation of outcomes at different follow-up time. Among available 269 patients who had follow-up data within 5 years and obtained functional independence at 90 days, 208 (77.3%) maintained good outcome, 19 (7.1%) developed disability (mRS 3–5) and 42 (15.6%) died at 5 years. Among available 189 patients with disability at 90 days, 53 (28.0%) obtained functional independence, 60 (31.7%) maintained and 76 (40.2%) died within 5 years. At 5 years, 261 patients (39.7%) had a good functional outcome, 317 patients (48.2%) died and 129 (28.2%) had stroke recurrence (Figure 3 and Supplementary Table 1).

### Secondary Outcomes

**FIGURE 4:**
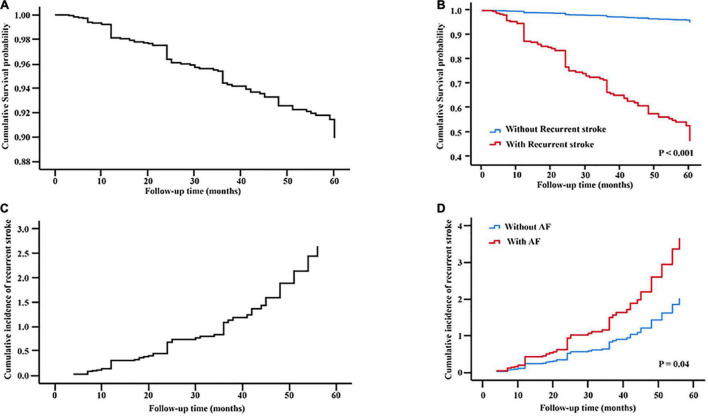
Multivariate Cox regression analysis. Cumulative survival probability were plotted according to **(A)** all patients and recurrent stroke presence **(B)** in all patients. Cumulative incidence curves of recurrence stroke were plotted according to **(C)** all patients and AF presence **(D)** in all patients. AF, atrial fibrillation.

The cumulative 90-day rate of death was 24.7% (199/807), and up to 48.2% (317/657) at 5-year follow-up (Supplementary Table 1 and Figure 3). Cox proportional hazards model showed that risk of death was especially high at 90 days, then patients who survived continued to die at a rate of approximately 2.8–5.8% per year for the next 5 years (Figure 4). Stroke recurrence was significantly correlated with mortality within 5 years (*P* < 0.001). In addition, atrial fibrillation was associated with the increase risk of stroke recurrence (*P* = 0.04). The relationship of different outcomes with age, mRS at 90 days and stroke recurrence was shown in Supplementary Figure 1.

### Predictors for Outcomes

After adjusting for major confounders, multivariate logistic regression analysis identified that younger age (OR: 0.96; 95% CI: 0.93–0.99; *P* = 0.009), lower mRS at 90 days (OR: 0.15; 95% CI: 0.10–0.23; *P* < 0.001) and without stroke recurrence (OR: 0.001; 95% CI: 0.000–0.006; *P* < 0.001) were independent predictors for good functional outcome. Advanced age (OR: 1.04; 95% CI: 1.01–1.07; *P* = 0.025), higher mRS at 90 days (OR: 4.07; 95% CI: 2.75–6.02; *P* < 0.001) and stroke recurrence (OR: 273.95; 95% CI: 74.72–1004.39; *P* < 0.001) were associated with increased mortality risk at 5 years. Furthermore, advanced age (OR: 1.06; 95% CI: 1.04–1.08; *P* < 0.001), atrial fibrillation (OR: 1.63; 95% CI: 1.02–2.60; *P* = 0.04) and lower mRS at 90 days (OR: 0.84; 95% CI: 0.73–0.98; *P* = 0.021) were significantly associated with stroke recurrence (Table 2 and Supplementary Table 2).

**TABLE 2:** Multivariable analysis: predictors of functional outcomes at 5 years follow up.

	mRS 0–2 at 5 years		Mortality at 5 years		Stroke recurrence at 5 years	
			
	Adjusted OR (95% CI)	*P*-value	Adjusted OR (95% CI)	*P*-value	Adjusted OR (95% CI)	*P*-value
Age (per year increase)	0.96 (0.93–0.99)	0.009	1.04 (1.01–1.07)	0.025	1.06 (1.04–1.08)	<0.001
Male	1.42 (0.64–3.16)	0.39	1.40 (0.65–2.97)	0.40	1.16 (0.70–1.94)	0.57
Smoking	1.09 (0.45–2.61)	0.86	0.45 (0.18–1.12)	0.087	0.71 (0.41–1.25)	0.23
Hypertension	1.14 (0.54–2.41)	0.74	0.86 (0.41–1.80)	0.68	1.17 (0.73–1.87)	0.53
Hyperlipidemia	0.50 (0.14–1.81)	0.29	1.67 (0.52–5.40)	0.39	1.46 (0.76–2.82)	0.26
Diabetes mellitus	0.94 (0.38–2.35)	0.90	1.22 (0.52–2.89)	0.65	1.24 (0.72–2.15)	0.44
Atrial fibrillation	0.96 (0.46–2.03)	0.92	1.45 (0.71–2.95)	0.31	1.63 (1.02–2.60)	0.04
mRS at 90 days (per 1 grade increase)	0.15 (0.10–0.23)	<0.001	4.07 (2.75–6.02)	<0.001	0.84 (0.73–0.98)	0.021
Stroke recurrence	0.001 (0.000–0.006)	<0.001	273.95 (74.72–1004.39)	<0.001	NA	NA

*OR, Odds ratio; CI, confidence interval; mRS, Modified Rankin Scale Score; NA, not applicable.*

## Discussion

This is a multicenter observational study of consecutive patients undergoing EVT for acute LVO in anterior circulation. Covering 28 comprehensive stroke centers in China, it gives an estimate on current practice of EVT outside from clinical trials. Focusing on the long-term functional outcome, our study revealed that the proportion of good outcome and excellent outcome at 5 years were comparable to the results at 90 days. A total of 208 patients (77.3%) sustained independent functional outcome at 5 years in 269 patients with good outcome at 90 days. Age, mRS at 90 days and stroke recurrence were independent factors of good outcome and mortality at 90 days. In addition, advanced age, lower mRS at 90 days and atrial fibrillation were significantly associated with stroke recurrence.

In a large national cohort of stroke patients from Australia and New Zealand, about half of patients hospitalized with a stroke survived at 5 years, and only a third at 10 years. Around one-fifth of patients had a stroke recurrence by 5 years, and 25% by 10 years (Peng et al., 2022). Compared with our study from Chinese population, an acute stroke reduced the life expectancy long-term functional outcomes. These findings reinforce the need for concerted efforts to improve acute stroke care and secondary prevention to advance long-term prognosis.

Reperfusion therapies clearly improve short-term functional outcome. However, long-term outcomes are poorly defined. Evidence is emerging that the efficacy of acute stroke treatments may persist over a few years. Improvement after intravenous thrombolysis is sustained over 3 years and EVT shows long-term efficacy up to 2 years (Schmitz et al., 2014; van den Berg et al., 2017). To our knowledge, this is by far the first multicenter large registry to evaluate the 5-year functional outcome of patients with acute LVO in anterior circulation who underwent EVT. Present results showed that short-term recovery in functional status could be sustained during the course of at least 5 years. Moreover, younger age, lower mRS at 90 days and absence of stroke recurrences were independent prognostic factors of functional outcome at 5-year follow up.

Our study showed that risk of death was especially high early after stroke: 24.7% of deaths occurred within the first 90 days after stroke. After 90 days, patients who survived continued to die at a rate of approximately 2.8–5.8% per year for the next 5 years. It seemed that most early deaths (first 90 days) after stroke were due to three main causes—the direct effects of extensive brain injury like brain edema; secondary complications of stroke like pneumonia, pulmonary embolism, and hemorrhagic transformation; and cardiac disease. On the other hand, the increased mortality risk at 5 years after stroke were contributed by increasing age, residual disability at 90 days and stroke recurrence.

Stroke recurrence is associated with higher mortality and poor functional recovery. Despite its common occurrence, long-term studies on stroke recurrence are sparse, especially in the patients with acute LVO who underwent EVT. Larger longitudinal studies showed cumulative stroke recurrence at 5 years after stroke varied between 16.2 and 48% in the general population (Meyer et al., 2015). In the present studies, the cumulative stroke recurrence at 5 years was 28.2%, and our results showed that the probability of stroke recurrence continued to increase gradually during the 5-year follow up. Predictors of long-term stroke recurrence are both modifiable and non-modifiable. A systematic review reported that increasing age, male sex, hypertension, diabetes mellitus, prior stroke, smoking, alcohol consumption, cardiac disease, stroke subtype, and stroke etiology were all associated with increased risk of late recurrences (Singh et al., 2018). In this study, the history of atrial fibrillation and the increasing age were independently predictors for stroke recurrence throughout the follow-up period up to 5 years. This was closely related to the current status of oral anticoagulation use among Chinese patients with atrial fibrillation. Studies from the China nationwide survey revealed that < 10% of hospitalized patients with atrial fibrillation were on warfarin therapy, and among community-based patients with atrial fibrillation, oral anticoagulation usage was even lower (2.7%) (Chang et al., 2016).

### Limitations

Our study has to deal with all the inherent limitations of a non-randomized observational registry study. The reasons for clinicians to select a specific treatment option are more complex than can be covered by the scope of an observational study. We also lacked a control group of patients who did not receive EVT for comparison. In addition, we didn’t use multiple imputation to perform sensitivity analysis as the information of many patients was lost in the 5-year period. Furthermore, we only evaluated the long-term functional outcome of LVO patients, but lacked the evaluation of quality of life. Finally, intravenous thrombolysis also represents a major selection bias in our study. Conversely, strengths of our study are—besides its large size—its investigator-initiated multicenter design, the assessment of imaging data by a blinded core laboratory, the monitoring of all participating centers to minimize reporting bias within the registry, and the focus on daily clinical practice for patients with acute anterior circulation LVO undergoing EVT, and despite its limitations, it still constitutes one of the best available data about LVO treatment. The data is not generalizable and only applicable to the Chinese population (recurrence risk/rates are different between the investigated populations). A lot of crucial outcome predictors are missing (treatment times, IVT, complications, reperfusion success etc.). The data is only applicable to patients with good pre stroke functional status. Selection criteria were not uniform and center dependent. Due to the retrospective selection of patients, there is selection bias, as it is very likely that only patients with good outcome are available for follow ups or agree to be available for long term follow ups.

## Conclusion

The beneficial effect of EVT in patients with AIS caused by LVO of the anterior circulation was sustained during the course of at least 5 years. Reducing the recurrence rate of stroke by anticoagulation for atrial fibrillation may be the crucial strategy to improve long-term patient outcome.

## Data Availability Statement

The raw data supporting the conclusions of this article will be made available by the authors, without undue reservation.

## Ethics Statement

The studies involving human participants were reviewed and approved by the ethics committee of the Xinqiao Hospital (Second Affiliated Hospital), Army Medical University Board (201308701). The patients/participants provided their written informed consent to participate in this study.

## Author Contributions

CG, JH, and WK interpreted the data and drafted the manuscript. WZ and QY contributed to the conception and design of the study. CL and JY did the statistical analyses. FL, SL, ZQ, ML, ZG, ZY, XH, SZ, WL, PZ, ZWa, YL, DX, YZ, SY, YW, JF, WH, and HL performed the acquisition, analysis, and interpretation of the data. JL, RL, CW, XF, MT, LW, XT, HP, ZWu, and GZ provided technical and material support, and made critical revision of the manuscript. All authors contributed to the article and approved the submitted version.

## Conflict of Interest

The authors declare that the research was conducted in the absence of any commercial or financial relationships that could be construed as a potential conflict of interest.

## Publisher’s Note

All claims expressed in this article are solely those of the authors and do not necessarily represent those of their affiliated organizations, or those of the publisher, the editors and the reviewers. Any product that may be evaluated in this article, or claim that may be made by its manufacturer, is not guaranteed or endorsed by the publisher.
