# Supplementary material for: Five-Year Outcomes After Endovascular Treatment for Large Vessel Occlusion Stroke
Source: Front Neurosci. 2022 Jul 13;16:920731. doi: 10.3389/fnins.2022.920731 (PMC9326078; doi:10.3389/fnins.2022.920731)
Supplement: Supplementary file 1 [file Table_1.docx]

Table S1: Outcomes at 90 days and 5 years

Table S2: Multivariable analysis: predictors of functional outcomes at 90 days and 5 years follow up

Table S1: Outcomes at 90 days and 5 years

|  | At 90 days (n=807) | At 5 years (n=657) |
| --- | --- | --- |
| mRS, median (IQR) | 3 (1–5) | 4 (1–6) |
| 0 | 66/807 (8.2) | 100/657 (15.2) |
| 1 | 152/807 (18.8) | 90/657 (13.7) |
| 2 | 131/807 (16.2) | 71/657 (10.8) |
| 3 | 90/807 (11.2) | 47/657 (7.2) |
| 4 | 115/807 (14.3) | 29/657 (4.4) |
| 5 | 54/807 (6.7) | 3 (0.5) |
| Mortality, n (%) | 199/807 (24.7) | 317/657 (48.2) |
| Stroke recurrence, n (%) | NA | 129/458 (28.2) |
| ICH | 118/807 (14.6) | NA |
| SAH | 19/807 (2.4) | NA |
| Hemicraniectomy | 64/807 (7.9) | NA |
| Arterial perforation | 8/807 (0.99) | NA |
| Systemic bleeding | 19/807 (2.4) | NA |

Abbreviations: mRS = Modified Rankin Scale Score; IQR= interquartile range; ICH = Intracerebral hemorrhage; SAH = subarachnoid-hemorrhage; NA = not applicable

Table S2: Multivariable analysis: predictors of functional outcomes at 90 days and 5 years follow up

|  | mRS score at 90 days | | mRS score at 5 years | |
| --- | --- | --- | --- | --- |
|  | Unadjusted OR (95% CI) | *P* value | Unadjusted OR (95% CI) | *P* value |
| mRS at discharged (per 1 grade increase) | 1.39 (1.37–1.42) | <0.001 | 1.29 (1.25–1.33) | <0.001 |

Abbreviations: OR = Odds ratio; CI = confidence interval; mRS = Modified Rankin Scale Score.
