# Supplementary material for: Five-Year Outcomes After Endovascular Treatment for Large Vessel Occlusion Stroke
Source: Front Neurosci. 2022 Jul 13;16:920731. doi: 10.3389/fnins.2022.920731 (PMC9326078; doi:10.3389/fnins.2022.920731)
Supplement: Supplementary file 2 [file Presentation_1.pdf]

Contents

Five-Year Outcome after Endovascular Treatment for Large Vessel Occlusion in Acute Ischemic Stroke (SUSTAIN study) Protocol

Protocol

Introduction

Stroke is one of the leading causes of disability and death worldwide[1]. Recently, several randomized clinical trials have proved the efficacy of endovascular treatment (EVT) for acute ischemic stroke (AIS) with larger vessel occlusion (LVO) in the anterior circulation at 3 months post randomisation[2-8]. However, results on long-term clinical outcome are still lacking, especially in a real-world setting.

With improvements in acute care, longer term outcomes will impact the organization of stroke care systems and the allocation of health care resources. This may be particularly true for stroke care organization in developing countries which have a higher stroke burden, but more limited health care resources.[9,10]

Therefore, we conducted this nationwide, observational study (SUSTAIN study) to assess the long-term outcomes after EVT for AIS due to LVO in the anterior circulation. This study has been registered on the website, Chinese Clinical Trial Registry (http://www.chictr.org.cn/index.aspx) (Registration No. ChiCTR1900020671).

Methods

Study design

The SUSTAIN study was an observational, nationwide registry of consecutive patients presented with acute anterior circulation stroke in whom an EVT procedure was attempted for LVO in China. The main purpose of the SUSTAIN study was to evaluate the long-term functional outcomes, stroke recurrence and mortality of patients with acute LVO in anterior circulation undergoing EVT. Participating centers were allowed to retrospectively include patients in the registry if patients were treated

within the past 3 years and if reliable clinical follow up information was available. To avoid selection bias, all participating centers were obliged to enroll all consecutive patients in the registry, including those being retrospectively enrolled in the database and those with unsuccessful treatment. All participating centers were monitored at the end of the recruitment period in order to avoid selection bias by cross-checking neurointerventional logs with the database. The study was designed by the principal investigators and experts in cerebrovascular diseases and interventional neuroradiology. The SUSTAIN study protocol has been approved by the ethics committee of the Xinqiao hospital, Army Medical University, Chongqing, China and each participating center.

Subjects selection

Inclusion criteria

Patients can be enrolled in the study if they fulfilled the following criteria:

1) age 18 years or older;

2) were diagnosed with AIS;

3) a LVO in anterior circulation confirmed by computed tomographic angiography, magnetic resonance angiography, or digital subtraction angiography;

4) initiation of intravenous recombinant tissue plasminogen activator (rt-PA) within 4.5 hours or intravenous urokinase within 6 hours of LVO;

5) underwent endovascular recanalization treatment;

6) informed consent.

Exclusion criteria

Patients will be excluded from the study in case of:

1) pre-existing dependency with a modified Rankin Scale (mRS) ≥ 2;

2) neuroimaging evidence of cerebral hemorrhage on presentation;

3) patients without follow-up information;

4) incomplete baseline critical data (e.g., imaging and time points)

5) currently in pregnant or lactating;

6) serious, advanced or terminal illness.

Participating center eligibility

To avoid selection bias, all centers were obliged to enter all consecutive patients in the study. To be fully eligible for participation in the cohort study, study site selection had to meet the following minimum criteria: all study centers were required to have performed at least 30 endovascular procedures annually, including at least 15 thrombectomy procedures; all intervention teams were certified interventionists for EVT on large artery occlusion.

Treatments

Patients underwent EVT which comprised mechanical thrombectomy, thromboaspiration, balloon dilation, stenting, intra-arterial thrombolysis, or various combinations of these approaches. The mechanical thrombectomy may consist of thrombus retraction, aspiration or use of a stent retriever device. Generally, solitaire FR (Medtronic Neurovascular, Irvine, CA), Trevo (Stryker neurovascular, Fremont, CA) or other newly developed devices approved by National Medical Products Administration (NMPA) were considered in the present study. Both intravenous sedation and general anesthesia can be considered to ensure the safety of patients. Re-occlusion often occurred after thrombectomy in atherosclerotic disease, therefore, rescue therapy including balloon dilation, stenting, intra-arterial thrombolysis, and glycoprotein IIb/IIIa inhibitor (GPI) might be utilized to retrieve recanalization. After recanalization of the target artery, most of the patients were transferred to the neuro-intensive care unit for at least 24 hours with their systolic blood pressure maintained at 120-140mmHg. Additionally, the patients who underwent extracranial or intracranial stent implantation were prescribed antithrombotic medication to prevent acute stent thrombosis. For the patients without prior intravenous thrombolysis (IVT), loading doses of clopidogrel (300 mg) and aspirin (300 mg) were given, or a low dose of GPI was bolus-injected intra-arterially and maintained for at

least 24 hours, while for those with prior IVT, clopidogrel (75 mg) and aspirin (100 mg) were given after 24 hours of IVT, then all the patients were given clopidogrel (75 mg/d) and aspirin (100 mg/d) for 1-3 months.

Patients received medical treatment, e.g. intravenous IVT with rt-PA or urokinase, antiplatelet drugs, systematic anticoagulation, or combinations of these medical treatments, as described in the guidelines for the management of AIS[11].

Data collection

We recorded patients' baseline characteristics, stroke risk factors, time of symptom onset, clinical presentation, stroke severity (assessed by the National Institutes of Health Stroke Scale (NIHSS), pretreatment and posttreatment imaging findings, type and timing of EVT, complications, presumed cause of stroke, cause of death, outcome measures, and safety measures. The presumed stroke causative mechanism was assessed based on the Trial of ORG 10172 in Acute Stroke Treatment (TOAST) classification.[12]

All angiography, computed tomographic and magnetic resonance imaging data were sent to the core laboratory, where evaluation was performed blinded to all clinical data. Initial vessel occlusion and recanalization were assessed with the modified thrombolysis in cerebral infarction (mTICI) score[13] Collateral status was evaluated using the American Society of Interventional and Therapeutic Neuroradiology/ Society of Interventional Radiology (ASITN/SIR) collateral grading system, and collateral scores were categorized into ASITN/SIR grades 0–1, 2, and 3–4.[14]The Alberta Stroke Program Early Computed Tomography Score (ASPECTS) was used to quantify the ischemic changes on baseline imaging.[15]

Follow-up

At 24 hours, a clinical examination including the National nstitutes of Health Stroke Scale (NIHSS) will be carried out. At 48 hours, patients will undergo CTA or MRA imaging to evaluate the recanalization rate and CT to assess the cerebral hemorrhage. At one-week, clinical status, NIHSS score, and adverse events will be reported. At 90 days, 1 year, 2 years, 3 years, 4 years and 5 years, mRS and stroke recurrence will be recorded.

Endpoints

Primary efficacy outcome

The primary outcome measure was the proportion of patients with good clinical outcome (mRS 0-2) at 5 years after stroke as assessed by trained local neurologists who were blinded to all clinical data. The mRS is a 7-level scale (range, 0 [no symptoms] to 6 [death]) for the assessment of neurologic functional disability.

Secondary efficacy outcomes

Secondary outcomes included the proportion of patients with good clinical outcome (mRS 0-2) at 90 days, excellent clinical outcome (mRS 0-1) at 90 days, excellent clinical outcome (mRS 0-1) at 5 years; death from any cause during the study period after stroke; risk of stroke recurrence that occurred at 90 days and 5 years after stroke. Symptomatic intracerebral hemorrhage (sICH) at 48 hours, procedure-related complications (e.g., arterial perforation, arterial dissection, and embolization in a previously uninvolved vascular territory), and serious adverse events. The technical efficacy outcomes regarding recanalization were substantial reperfusion, as assessed by means of catheter angiography in the EVT group and defined as a modified Treatment in Cerebral Infarction score of 2b (50 to 99% reperfusion) or 3 (complete reperfusion). Stroke was defined according to the World Health Organization criteria as “rapidly developing symptoms and/or signs of focal, and at times global, loss of cerebral function, with symptoms lasting more than 24 hours or leading to death with

no apparent cause other than that of vascular origin."[16] Although the definition of recurrent stroke was not strictly identical in each study, in practice, we used the above definition to define recurrent stroke as stroke and met the following criteria: there was clinical evidence of the sudden onset of a new focal neurological deficit with no apparent cause other than that of vascular origin (i.e., the deficit could not be ascribed to an inter-current acute illness, epileptic seizure, or toxic effect) occurring at any time after the index stroke; or there was clinical evidence of the sudden onset of an exacerbation of a previous focal neurological deficit with no apparent cause other than that of vascular origin.[17-19]

Blinding

All those involved in the subsequent clinical and imaging assessment of outcomes will be blinded to treatment allocation.

Imaging core laboratory

Centralized imaging core labs will be used in this study to provide consistent evaluation of images. The imaging core laboratory evaluated the findings on baseline non-contrast CT for the posterior circulation- Alberta Stroke Program Early CT Score (pc-ASPECTS), baseline vessel imaging (CTA, MRA, or DSA) for the location of the occlusion, angiographic outcomes on DSA imaging for technical efficacy outcomes regarding recanalization, follow-up CTA or MRA within 48 hours for vessel recanalization, and the follow-up CT for the presence of intracerebral hemorrhage. All neuroimaging studies were evaluated by two neuroradiologists independently with blindness of the treatment allocation, clinical data, and outcomes. For cases with disagreement, decisions were made by the third experienced neuroradiologist.

Clinical events committee

The clinical events committee will be comprised of three expert physicians who are independent of the investigational sites. This committee will be responsible for the review and validation of all complications that occur over the course of the study and

the subsequent classification of these complications as related to the device or procedure.

Members of the clinical events committee will review all complications and adjudicate them as defined in the adverse event section in the clinical events committee manual of operations. The clinical events committee can request additional source documentation and any imaging obtained in support of the adverse event to assist with adjudication.

Statistical analysis

Statistical analyses were performed using SPSS version 23 (IBM Corp., Armonk, NY) and STATA version 15.2 (Stata Corp LLC, TX). Mean (standard deviation) or median (interquartile range [IQR]) was used to describe patients' characteristics for continuous variables. Categorical variables were described as frequencies (percentages). Student's tests or Mann–Whitney U tests were used to compare the differences between continuous variables. Trend tests or Fisher exact tests were used to compare categorical as appropriate. Significant variables with $P<0.05$ on univariable analysis were entered into multivariable analysis. Binary logistic regression with the enter method (ordinal variables were compared using simple [first] method) was used to identify independent predictors for poor functional outcome. Kaplan–Meier analysis was used to reflect the long-term survival and stroke recurrence probability of LVO patients treated with EVT. As stated earlier, patients who did not consent to the extended follow-up may introduce an important selection bias. To gain as much information as (legally) possible on the clinical status of these patients, a waiver from the Institutional Review Board was obtained to assess the vital

status of these patients at five years of follow-up. This information will be used for the survival analysis as well as for the sensitivity analyses of the outcome.

Discussion

Stroke patients ofen have signifcant disability, incurring a large societal monetary burden [1]. Despite multiple RCTs and meta-analyses highlighting the safety and efcacy of endovascular thrombectomy at a standard 3-month period, the long-term outcome has received less attention. To this day, long-term clinical outcomes were only available for 1-year follow-up in IMS III and REVASCAT and at 2 years in MR CLEAN.[20-22] A meta-analysis pooled data from the aforementioned three trials demonstrate that, compared to medical management, endovascular therapy results in favorable functional independence, health-related quality of life, and cognitive function at long-term follow-up for patients with AIS.[23] However, few studies have investigated 5 or more years outcomes for AIS treated with EVT. In the current study we extend the follow-up duration to five years after stroke to estimate the effect of EVT on functional outcome over the longer term. Secondary objectives include the effect of EVT on stroke recurrence and mortality during five years.

During longer term follow-up studies, loss to follow-up is a well-known phenomenon, resulting in smaller sample sizes. Furthermore, loss to follow-up may cause serious attrition bias. Both these problems may play an important role in our study. Therefore, we will provide clear information on the flow of patients through the study and

differences in baseline and measured variables, and we will perform additional sensitivity analyses for different scenarios.

The results of our study will provide information on the five-year clinical effectiveness of EVT for patients with AIS due to large vessel occlusion in the anterior circulation. The benefit of EVT on short-term disability might translate into longer term improvements in survival and functional status, which may be useful for individual treatment decisions and estimates of cost-effectiveness. Subsequently, it will have important additional value concerning implementation of EVT all over the world.

Study personnel

Principal Investigator

Qingwu Yang, MD; Wenjie Zi,

Executive committee

Weidong Luo, Shuai Liu, Luming Chen, Dongjing Xie, Fei Gao, Jiaxing Song, Chenhao Zhao, Junjie Yuan, Jiacheng Huang, Hongfei Sang, lingyu Li,Hansheng Liu,

Steering committee

Wenjie Zi, MD; Fengli Li, Zhongming Qiu, MD, MD; Qingwu Yang, MD

Clinical events committee

Fan Deng, Dan Liu, Shengya Zeng

Imaging assessment committee

Dong Zhang, Hua Yang, Fajin Lv

Outcome assessment committee

Zhangbao Guo,Min Zhang, Jiaming Cao, Zhizhong Yan, Shuai Zhang, Li Wang, Dongzhang Xue, Fanghui Bai, Kuihua Wang, Rongzhong Li, Tieqiao Feng, Zhen Wang, Wentong Ling, Kui Lu, Hongliang Zeng, Jianzhou Wu, Xuan Liu, Zetao Shao, Yong Liu, Shu Yang, Haitao Guan, Qiang Shi, Renliang Meng, Peiyang Zhou, Yaoyi Zhong, Jiayang Fang,Wenguo Huang, Huihui Liu,Jun Luo,Changming Wen,Xinmin Fu,Mingyi Tu,Xiguang Tian, Huiyuan Peng,Zhilin Wu,Guoyong Zeng. .

PhD-students and study coordinators

Chengsong Yue, Jun Yang, Weidong Luo, Shuai Liu, Pengxiao Yu, Luming Chen, Dongjing Xie, Fei Gao, Jiaxing Song, Chenhao Zhao, Junjie Yuan, Guoqiang Yang, Jiacheng Huang, Hongfei Sang, Yonggang Hao, Feng Peng, Xiaoyun Liu, Jie Yang, Department of Neurology, Xinqiao Hospital and The Second Affiliated Hospital, Army Medical University (Third Military Medical University), Chongqing, China.

Study statisticians

Rufu Xu (chairman), Dong Yi, Ling Liu, Mengjie Lu

Reference

1. Krishnamurthi RV, Feigin VL, Forouzanfar MH, et al. Global and regional burden of first-ever ischaemic and haemorrhagic stroke during 1990–2010: findings from the Global Burden of Disease Study 2010. *The Lancet Global Health.* 2013;1(5):e259-e281.doi:10.1016/s2214-109x(13)70089-5
2. Berkhemer OA, Fransen PS, Beumer D, et al. A randomized trial of intraarterial treatment for acute ischemic stroke. *N Engl J Med.* 2015;372(1):11-20.doi:10.1056/NEJMoa1411587
3. Goyal M, Demchuk AM, Menon BK, et al. Randomized Assessment of Rapid

Endovascular Treatment of Ischemic Stroke. *New England Journal of Medicine.* 2015;372(11):1019-1030.doi:10.1056/NEJMoa1414905

4. Campbell BCV, Mitchell PJ, Kleinig TJ, et al. Endovascular Therapy for Ischemic Stroke with Perfusion-Imaging Selection. *New England Journal of Medicine.* 2015;372(11):1009-1018.doi:10.1056/NEJMoa1414792
5. Saver JL, Goyal M, Bonafe A, et al. Stent-retriever thrombectomy after intravenous t-PA vs. t-PA alone in stroke. *N Engl J Med.* 2015;372(24):2285-2295.doi:10.1056/NEJMoa1415061
6. Jovin TG, Chamorro A, Cobo E, et al. Thrombectomy within 8 hours after symptom onset in ischemic stroke. *N Engl J Med.* 2015;372(24):2296-2306.doi:10.1056/NEJMoa1503780
7. Bracard S, Ducrocq X, Mas JL, et al. Mechanical thrombectomy after intravenous alteplase versus alteplase alone after stroke (THRACE): a randomised controlled trial. *The Lancet Neurology.* 2016;15(11):1138-1147.doi:10.1016/s1474-4422(16)30177-6
8. Martins SO, Mont'Alverne F, Rebello LC, et al. Thrombectomy for Stroke in the Public Health Care System of Brazil. *N Engl J Med.* 2020;382(24):2316-2326.doi:10.1056/NEJMoa2000120
9. Lozano R, Naghavi M, Foreman K, et al. Global and regional mortality from 235 causes of death for 20 age groups in 1990 and 2010: a systematic analysis for the Global Burden of Disease Study 2010. *The Lancet.* 2012;380(9859):2095-2128.doi:10.1016/s0140-6736(12)61728-0
10. Murray CJL, Vos T, Lozano R, et al. Disability-adjusted life years (DALYs) for 291 diseases and injuries in 21 regions, 1990–2010: a systematic analysis for the Global Burden of Disease Study 2010. *The Lancet.* 2012;380(9859):2197-2223.doi:10.1016/s0140-6736(12)61689-4
11. Powers WJ, Derdeyn CP, Biller J, et al. 2015 American Heart Association/American Stroke Association Focused Update of the 2013 Guidelines for the Early Management of Patients With Acute Ischemic Stroke Regarding Endovascular Treatment: A Guideline for Healthcare Professionals

From the American Heart Association/American Stroke Association. *Stroke.* 2015;46(10):3020-3035.doi:10.1161/STR.0000000000000074

12. Adams HP, Jr., Bendixen BH, Kappelle LJ, et al. Classification of subtype of acute ischemic stroke. Definitions for use in a multicenter clinical trial. TOAST. Trial of Org 10172 in Acute Stroke Treatment. *Stroke.* 1993;24(1):35-41.doi:10.1161/01.str.24.1.35

13. Tomsick T, Broderick J, Carrozella J, et al. Revascularization results in the Interventional Management of Stroke II trial. *AJNR Am J Neuroradiol.* 2008;29(3):582-587.doi:10.3174/ajnr.A0843

14. Higashida RT, Furlan AJ, Roberts H, et al. Trial design and reporting standards for intra-arterial cerebral thrombolysis for acute ischemic stroke. *Stroke.* 2003;34(8):e109-137.doi:10.1161/01.STR.0000082721.62796.09

15. Puetz V, Sylaja PN, Coutts SB, et al. Extent of hypoattenuation on CT angiography source images predicts functional outcome in patients with basilar artery occlusion. *Stroke.* 2008;39(9):2485-2490.doi:10.1161/STROKEAHA.107.511162

16. Hatano S. Experience from a multicentre stroke register: a preliminary report. *Bull World Health Organ.* 1976;54(5):541-553. Published 1976/01/01.

17. Dennis MS, Burn JP, Sandercock PA, Bamford JM, Wade DT, Warlow CP. Long-term survival after first-ever stroke: the Oxfordshire Community Stroke Project. *Stroke.* 1993;24(6):796-800.doi:10.1161/01.str.24.6.796

18. Burn J, Dennis M, Bamford J, Sandercock P, Wade D, Warlow C. Long-term risk of recurrent stroke after a first-ever stroke. The Oxfordshire Community Stroke Project. *Stroke.* 1994;25(2):333-337.doi:10.1161/01.str.25.2.333

19. Hankey GJ, Jamrozik K, Broadhurst RJ, et al. Long-term risk of first recurrent stroke in the Perth Community Stroke Study. *Stroke.* 1998;29(12):2491-2500.doi:10.1161/01.str.29.12.2491

20. Palesch YY, Yeatts SD, Tomsick TA, et al. Twelve-Month Clinical and Quality-of-Life Outcomes in the Interventional Management of Stroke III Trial. *Stroke.* 2015;46(5):1321-1327.doi:10.1161/STROKEAHA.115.009180

21. Dávalos A, Cobo E, Molina CA, et al. Safety and efficacy of thrombectomy in acute ischaemic stroke (REVASCAT): 1-year follow-up of a randomised open-label trial. *The Lancet Neurology.* 2017;16(5):369-376.doi:10.1016/s1474-4422(17)30047-9
22. van den Berg LA, Dijkgraaf MG, Berkhemer OA, et al. Two-Year Outcome after Endovascular Treatment for Acute Ischemic Stroke. *N Engl J Med.* 2017;376(14):1341-1349.doi:10.1056/NEJMoa1612136
23. McCarthy DJ, Diaz A, Sheinberg DL, et al. Long-Term Outcomes of Mechanical Thrombectomy for Stroke: A Meta-Analysis. *ScientificWorldJournal.* 2019;2019:7403104.doi:10.1155/2019/7403104
