# Supplementary material for: Five-Year Outcomes After Endovascular Treatment for Large Vessel Occlusion Stroke
Source: Front Neurosci. 2022 Jul 13;16:920731. doi: 10.3389/fnins.2022.920731 (PMC9326078; doi:10.3389/fnins.2022.920731)
Supplement: Supplementary file 3 [file Image_1.pdf]

Figure S1 Margin effect. Relationship of different outcomes among age, mRS at 90 days and stroke recurrence at 5 years.

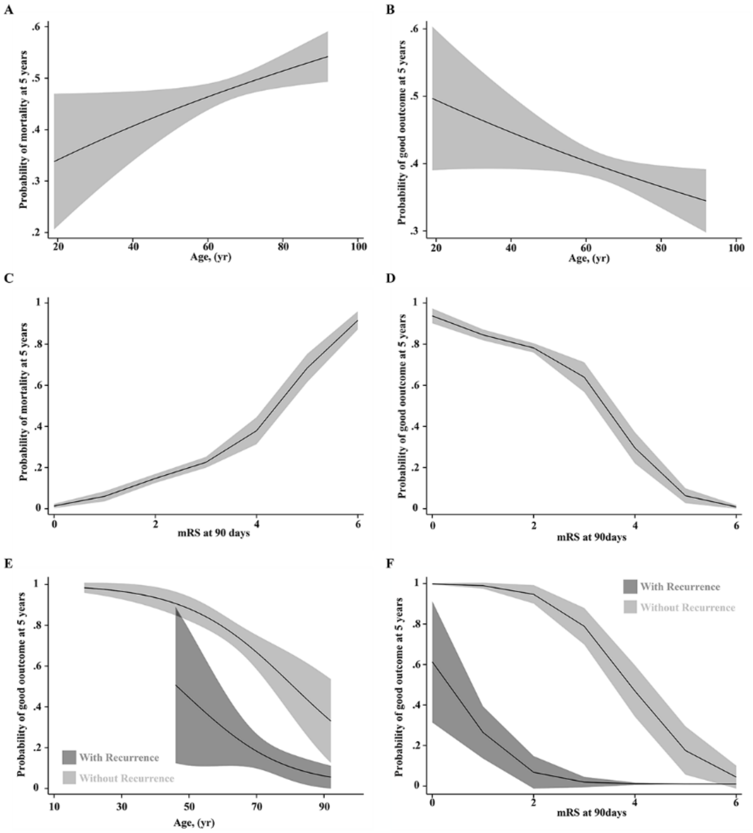

Figure S1 Margin effect. Relationship of different outcomes among age, mRS at 90 days and stroke recurrence at 5 years. Curves show that with the increases of age, the predicted mortality is increasing (A) but the predicted good outcome is decreasing (B). Figure4C: With the increases of age, good outcome probability is lower in the with recurrence group than the group with recurrence. Curves show that with the increases of mRS at 90 days, the predicted mortality is increasing (D) but the predicted good outcome is decreasing (E). Figure4F: With the increases of mRS at 90 days, good outcome probability is lower in the recurrence group than the group without recurrence.
